# Evaluating the impact of a medical school cohort sexual health course on knowledge, counseling skills and sexual attitude change

**DOI:** 10.1186/s12909-020-02482-x

**Published:** 2021-01-08

**Authors:** Michael W. Ross, Carey Roth Bayer, Alan Shindel, Eli Coleman

**Affiliations:** 1 Program in Human Sexuality, University of Minnesota Medical School, Minneapolis, MN USA; 2 Departments of Community Health and Preventive Medicine/Medical Education, Morehouse School of Medicine, Atlanta, GA USA; 3 Department of Urology, School of Medicine, University of California at San Francisco, San Francisco, CA USA

## Abstract

**Background:**

Sexual health is generally considered an integral part of medical and allied healthcare professional training. However, many medical schools do not offer this as a mandatory curriculum, or minimize it. Sexual health as an academic area was introduced in the 1970s, but there have been few cohort evaluations of its impact. This was limited by the availability of few psychometric scales for evaluation. We evaluated the full, mandatory, sexual health course in year 1 medicine at a large state university in the Midwest US, including the course with lectures; panels and tutorials; a video app to give students feedback on their sexual history taking skills; and a 3-station sexual history OSCE at the end of the course.

**Results:**

Seventy-four medical students (43% of the course cohort) volunteered, for an incentive, to complete evaluation materials pre- and post-course. We used the Sexual Health Education for Professionals Scale (SHEPS), designed and with appropriate psychometric standardization for such evaluation. The SHEPS data covers 7-point Likert scale ratings of 37 patient situations, asking first how well the student could communicate with such a patient, and on the second part how much knowledge they have to care for such a patient. The third subscale examines personal sexual attitudes and beliefs. Data indicated that the matched pretest-posttest ratings for skills and knowledge were all statistically significant and with very large effect sizes. Few of the attitude subscale items were significant and if so, had small effect sizes. Sexual attitudes and beliefs may be well-formed before entry into medical school, and sexual health teaching and learning has minimal effect on sexual attitudes in this US sample. However, using the 3 sexuality OSCE cases scores as outcomes, two of the 26 attitude-belief items predicted > 24% of the variance.

**Conclusions:**

The sexual health course produced major changes in Communications with patients sexual health skills and Knowledge of sexual health, but little change in personal Attitudes about sexuality. These data suggest that personal attitude change is not essential for teaching US medical students to learn about sexual health and sexual function and dysfunction, and comfortably take a comprehensive sexual history.

## Introduction

There is widespread support for including sexual health as an integral part of the basic curriculum for medical, physician assistant (PA) and nursing students [1–5]. Despite such agreement, a 2003 study [6] reported that in valid responses from 101 of 141 U.S. and Canadian medical schools surveyed, over half offered only 3–10 h for the sexual health curriculum and just a third offered 11 or more hours. Twenty percent of medical schools in the U.S. were reported as not requiring mandatory sexual health courses in the curriculum. A later study [7] found that 44% of US medical schools lacked formal sexual health curricula. In nursing schools, only 1 in 6 instructors thought that their students were prepared to deal with sexuality issues [8]. The lack of robust sexual health training for nursing and medical students may be due to a variety of factors, including competition for space in the curriculum, lack of appropriately trained faculty, and failure to recognize the importance of sexuality in clinical practice. Sometimes it is relegated as an elective area.

There is a paucity of well-evaluated curricula and standardized psychometric instruments to demonstrate the impact of sexual health courses on student knowledge and skills in the area. Evaluation of academic courses should cover the educational domains of teaching. Bloom’s Taxonomy of Educational Objectives [9] covers the domains of cognitive aspects (knowledge), affective components (comfort and feelings about the subject matter, traditionally addressed by Sexual Attitude Reassessment [SAR] seminars), and psychomotor aspects, more broadly conceptualized as the skills needed to perform a history-taking or a physical examination. Parish and Clayton [10] similarly identify the key domains of model sexual health curricula as attitudes, knowledge, and skills. Traditionally, attitudes toward sexual health issues have been seen as both precursors to, and products of, training in sexual health or other sexuality education, but it is difficult in cross-sectional studies to identify the contribution of attitudes about sexuality to affect, knowledge and skills.

There are few comprehensive evaluations of sexual health programs in medical schools in the literature. Marcotte and Logan [11] reviewed the data in the mid-1970s and found that SAR workshops which were used to expose students to differing sexual attitudes, showed significant increases in sexual knowledge and positive attitudes toward sexual behaviors following a 2-day SAR in male medical students in 1971. One study on medical students in California showed no changes in attitudes from baseline after a sex education course [12], while courses at the University of Minnesota and the University of South Carolina showed significant positive improvement over time [13, 14]. These early studies used the Sexual Attitudes and Behaviors Scale and the Sexual Knowledge and Attitude Test (SKAT) [15, 16] developed by Leif, first published in 1964 and copyrighted.

Garrard et al. [14] carried out a longitudinal study with the SKAT, in the 1970s at the University of Minnesota, and found significant increases in tolerant attitudes and knowledge which persisted at 12 months. Schnarch and Jones [17] also used the SKAT to evaluate the sex education course at Louisiana State University for 2nd year medical students, using final year medical students who had not attended the course as controls. They found that more liberal (positive) attitudes toward masturbation, abortion, and homosexuality and heterosexuality, and recognition of sexual myths, were significant in those who had attended the course compared with baseline, but not significant compared with the final year students who had not taken the course.

Twenty years later, Leiblum [18] carried out a largely qualitative cross-sectional analysis of the human sexuality program at Robert Wood Johnson Medical School that was designed for medical students, PAs, nurses/midwives, graduate nurses, MPH students, and other health care professionals. She noted high scores on the extent to which the course increased comfort levels talking about sexuality, understanding their own values, increasing their tolerance for the variety of human behavior, and basic knowledge of sexual practices and behaviors. In the same program, Rosen et al. [19] evaluated a half-day intensive workshop for residents who had not studied sexual health courses at medical school. Using a pretest-posttest design, 67% the 34 participants indicated that they had attained a greater awareness of sexual problems, and 52% reported that the workshop had helped them a great deal in developing comfort and skill in sexual history taking. While textbooks have been published on sexual history taking [20, 21], evaluation of the impacts of sexual health courses remains rare.

Evaluation of contemporary sexual health courses requires relevant and standardized evaluation instruments. The SHEPS (Sexual Health Education in Professionals Scale) was developed by Bayer and Shindel in the late 2010s [22] and was first evaluated in both the U.S. and East Africa [23, 24]. A central characteristic of the SHEPS is that instead of looking at general estimates of sexual health knowledge, it focuses on 37 specific provider knowledge questions where the *stem* was about providing sexuality services, based on the *item* of specific patient types (e.g. Q3, “A pubescent person, i.e. body changes with puberty, becoming sexually active, decision making”; Q29, “A person who desires contraception”). These questions are first scored by the student on a 7-point Likert scale on ability to communicate/assess/discuss the topic with a patient (*sexuality communication skills*), and then on confidence that one has the *knowledge* to care for patients with such a concern. Finally, there is a 26 item attitude scale on contemporary sexual attitudes and beliefs. The SHEPS was designed specifically to evaluate educational programs for healthcare providers. We hypothesized that the SHEPS would measure, from baseline to completion of the course, significant impacts on sexual health communication skills, knowledge, and attitudes in first year medical students. In arguing for more sexual health courses in US medical schools, evidence of a measurable impact is essential. Given the paucity of data on sexual health OSCEs, we were also interested in the relationship of the formal curriculum to the OSCEs.

## Method

### The course

**Fig. 1:**
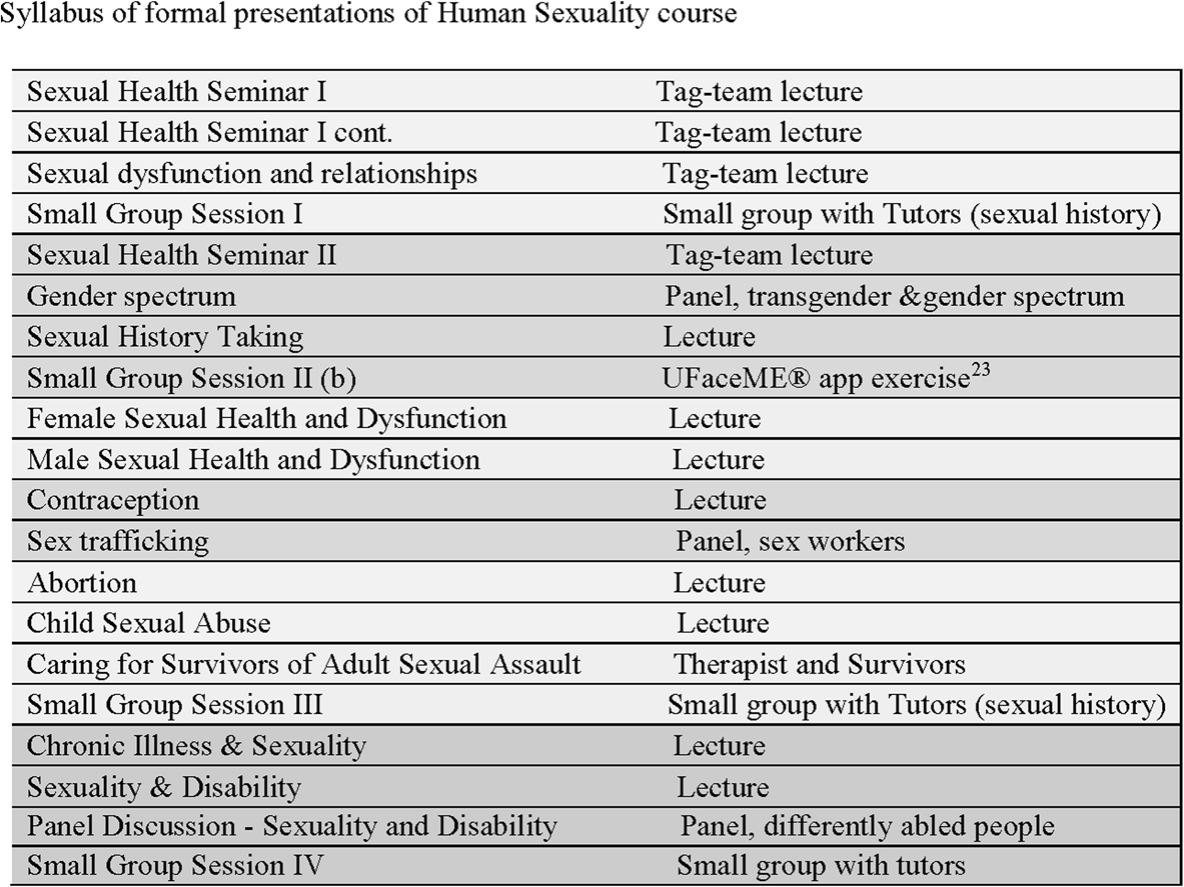
Syllabus of formal presentations of Human Sexuality course

We evaluated the mandatory sexual health course in year one at a major US medical school in 2019. This comprised (Fig. 1) 3 components: (1) 20 h of didactic lectures, panels, and tutorials, including two tutorials on sexual history taking, (2) a tutored app which gave students the opportunity to watch themselves taking a sexual history (based on a case history), with a colleague playing the “patient”, then reversing roles and getting feedback by rating each other in the exercise [25], and (3) the final exercise, 3 sexual health OSCE stations. The 3 OSCE stations involved a female standardized patient (SP); a male SP; and a transgender assigned male at birth SP; all with appropriate provided case histories. The University of Minnesota *M Simulation* Center designs and delivers simulated training experiences for all health sciences learners. The case histories for SP training and support were provided by the Program in Human Sexuality and the School of Nursing. We used the SHEPS for pre- and post-tests.

### Procedure

The entire first year (*N* = 174) of the medical course at the University of Minnesota was invited to engage in the study after the first semester of first year (December). Invitations were to participate by electronically filling out the SHEPS and demographic data (February), with an incentive of $US50 each ($100 total) for pretest and post-test. Post-test was in the week immediately following the end of the summer semester, a few days after the final exam (OSCE, end of June). Pre- and post-tests were anonymous, matched by a student-chosen code usually consisting of initials and a number. After data entry, matching, and cleaning, 6 questionnaires were eliminated: 80 pre-tests and 74 post-tests were completed and matched, leaving a sample size of 74. The period between baseline tests and the course was a semester, approximately 16 weeks. No sexual health material was taught before the pre-test, and the Sexual Health course ran for 20 h spread over 6 weeks, with the OSCE in the 7th week. The three (unknown patient sexual history, 8 min per case) OSCE scores consisted of SP ratings of the student “provider” on how engaged the SP felt, how responsive the provider seemed, how well listened to the SP felt, how at ease with the provider the SP felt, how engaged the provider seemed, how at ease the provider seemed, how on track the interview seemed, how much information the provider shared, and how much the provider encouraged sharing on the part of the SP, scored on a 5-point Likert scale where 1 = poor and 5 = excellent. The OSCE, using experienced SPs who had played sexual health cases for two previous years, was provided as the final practice evaluation and the score was not used for grading, but for research evaluation purposes. The study was approved by the University of Minnesota IRB, study number 00004500. Written informed consent was obtained after reading a plain-language description of the study.

### Analysis

Data were analyzed in SPSS version 26 (SPSS Inc., Chicago, Illinois). For the Attitudes scale, since it is composed of both positive and negative items, the negative items were reversed for Attitude scale analyses, with a high score indicating conservatism and a low score liberalism. *T*-tests were computed between pre- and post-test items, followed by effect size (Cohen’s *d*) calculations, for significant results, using the SocSciStatistics online calculator [26] (Tables 2 and 3). Difference scores on all items were calculated by subtracting the Pre-test item score from the Post-test item score. The OSCE scores were factor analyzed (Principal Components, Direct Oblimin rotation, Δ = 0) and only one factor with an eigenvalue > 1 emerged. Consequently, the scores were summed to form an OSCE total (α = 0.95). Correlations between OSCE total scores and SHEPS item difference scores were conducted using Pearson’s *r* (not shown). All significance tests were at *p* < 0.05, two-tailed.

## Results

### Sample

Response was 74/174, 42.5%. The sample characteristics are displayed in Table 1. Data comparing the gender of the sample with the medical class indicated that the sample was composed of more women (66%) than men (34%), not significantly different from the sex breakdown of the class (Yates corrected χ^2^ = 1.68, df = 1, *p* = 0.19). Median age for the sample and for the larger class was 24. Half of the sample (39, 52.7%) reported some formal sexual health education prior to medical school. Nearly all of the sample (68, 91.9%) reported that they knew someone who was a sexual minority (LGBTQ).

**Table 1:** Sample Characteristics (n, %)

Age	
Median	24
range	21–31
Gender	
Male	25 (34%)
Female	48 (65%)
Place of birth	
Minnesota	35 (42.7%)
Other	U.S. 42 (58.5%)
Overseas	5 (6.1%)
Most common projected specialty	
Family medicine	11 (14.9%)
Pediatrics	7 (9.5%)
Emergency medicine	7 (9.5%)
Surgery	7 (9.5%)
Internal medicine	5 (6.8%)
Ob/Gyn	4 (5.4%)
Sexual orientation	
Heterosexual	61 (82.4%)
Bisexual	3(4.1%)
Lesbian	3(4.1%)
Unsure	3(4.1%)
Prefer not to answer	4 (5.4%)
Ethnicity	
Hispanic/Latino/Chicano	2 (2.7%)
Prefer not to answer	2 (2.7%)
Race	
Asian/Pacific islander	9 (12.2%)
Black/African American	5 (6.8%)
Multiracial	3 (4.1%)
White/Caucasian	54 (73.0%)
Other/Prefer not to answer	3 (4.1)
Relationship status	
Single	18 (24.3%)
Casual dating	2 (2.7%)
Relationship	48 (64.9)
Married	5 (6.8%)
Prefer not to answer	1 (1.4%)
Know someone close who is LGBT	
Yes	68 (91.9%)
No	5 (6.8%)
Unsure	1 (1.4%)
Received formal education in sexuality before medical school	
Yes	39 (52.7%)
No	31 ((41.9%)
Don’t know	5 (6.8%)
Age group at which received formal sexuality education	
6–12	18 (46.2%)
13–18	16 ((41.2)
18+	4 (10.3%)
Don’t know	1 (2.6%)

**Fig. 2:**
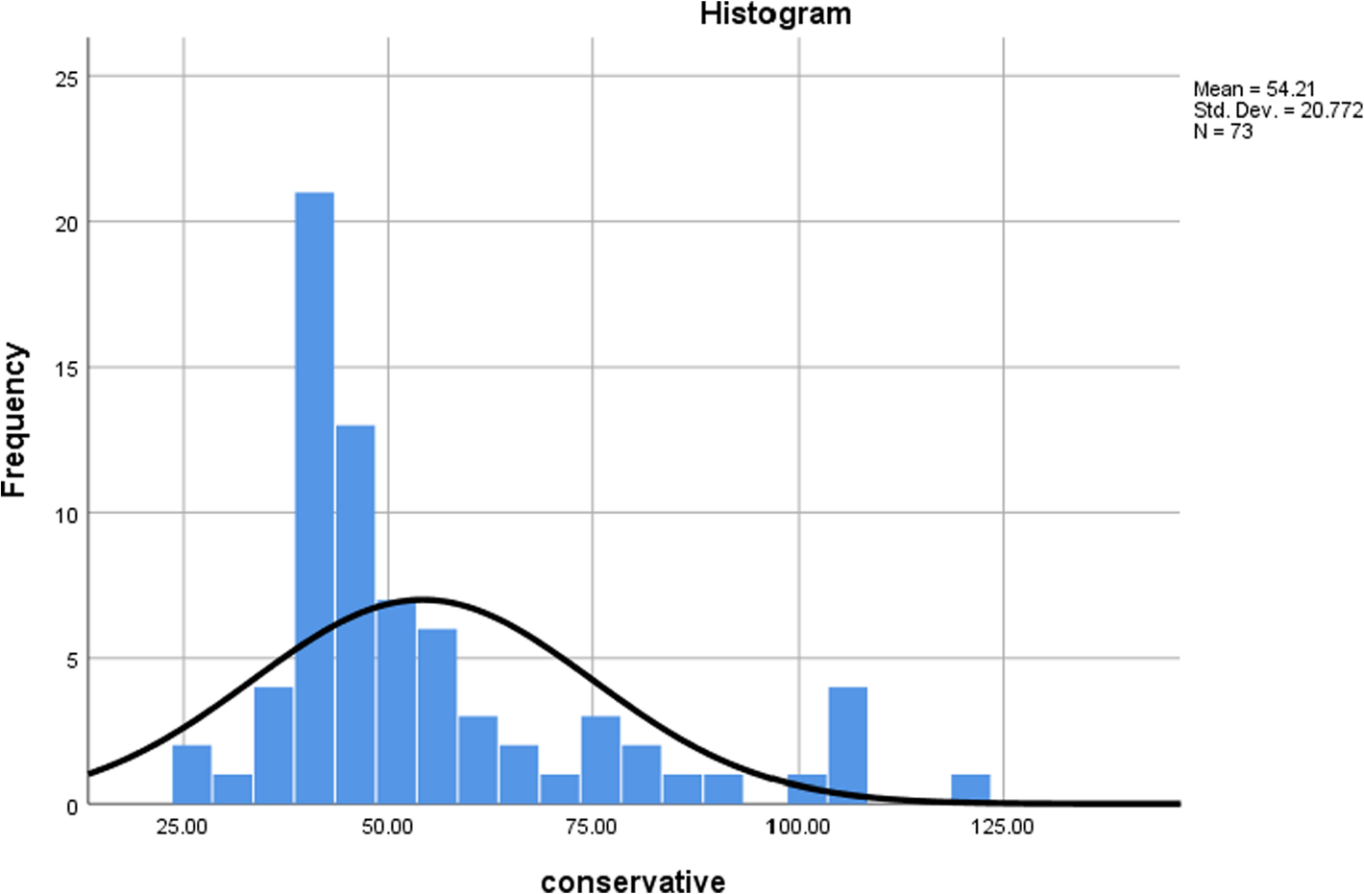
Distribution of Liberal (low)-Conservative (high) Post-test Attitudes

The SHEPS took about 20 min to complete. Data (Table 2) for the Communications skills subscale were significant between pretest and post-test at high levels of significance, and effect size calculations (Cohen’s *d* effect size: 0.20 small; 0.50 medium; 0.80 large; 1.40 huge) indicated 29 large and 1 huge effect sizes. Similar significance and effect sizes occurred in the Knowledge subscale, with 26 large and 9 huge effect sizes (Table 2). However, there were only 7 significant differences in the Attitudes subscale, and those with only 5 small and 1 medium effect sizes (Table 3). In a stepwise linear regression of the 6 post-test Attitude scale (attitudes within the week following the OSCE), items which significantly predicted the total OSCE score at *p* < .05 were identified. Two variables (“Sex is not an issue that physicians should deal with in their practices” (Yes), standardized β = 0.36, *p* = .000, and “Healthy women always have a lower sex drive than men” (Yes), standardized β = 0.26, *p* = .000) entered into the equation. The model including both these variables was significant (F = 11.84, *df* = 2, *p* = .000), *r* = 0.49, adjusted *r*^2^ = 0.24, accounting for 24% of the variance in the SP total OSCE score. Correlation of the OSCE total with the attitude scale total was *r*_*s*_ = − 0.15, *p* = 0.21: Attitude scale scores were approximately normally distributed but skewed toward the liberal (right) end of the continuum (see Fig. 2 post-test).

**Table 2:** Means and SDs Means and SDs on the Communications Skills and Knowledge Subscales of the SHEPS

	How confident are you in your ABILITY TO COMMUNICATE/ASSESS/DISCUSS sexuality and sexuality-related topics with…						Do you feel confident that you have the KNOWLEDGE TO CARE FOR PATIENTS when discussing sexuality and sexuality-related topics in…					
SHEPS Communications Skills and Knowledge Items	SHEPS Skills Items^a^						SHEPS Knowledge Items^a^					
	Mpre^b^	SDpre	Mpost	SDpost	*p*	*d*^c^	Mpre^b^	SDpre	Mpost	SDpost	*p*	*d*^c^
… the parents of a fetus or newborn with a disorder of sex development (e.g., ambiguous genitalia)	4.57	1.84	3.50	1.53	.000	0.63	5.56	2.14	3.68	1.67	.000	0.98
… a pre-pubescent child (i.e., masturbation, genital exploration of self and other children, questions about sex, “birds and the bees”)	4.62	1.59	2.82	1.35	.000	1.22	4.56	2.19	2.55	1.37	.000	1.10
… a pubescent person (i.e., body changes with puberty, becoming sexually active, decision making)	3.89	1.53	2.27	1.11	.000	1.21	4.12	2.20	2.21	1.27	.000	1.07
… a young (18–40 years) adult, (i.e., promoting sexual wellness)	2.97	1.28	1.77	0.93	.000	1.07	3.59	2.19	1.93	1.17	.000	0.95
… a middle aged (41–65 years) adult (i.e., promoting sexual wellness)	3.74	1.66	2.00	0.92	.000	1.30	4.12	2.30	2.15	1.29	.000	1.07
… an older (> 65 years) adult (i.e., changes in sexuality with aging)	4.55	1.71	2.47	1.02	.000	1.48	4.96	2.16	2.63	1.43	.000	1.27
… a person with mental disability (e.g., Downs Syndrome, schizophrenia, traumatic brain injury)	4.95	2.24	3.36	1.45	.000	0.74	5.68	1.98	3.49	1.45	.000	1.26
… a person with physical disability (e.g., cerebral palsy, spinal cord injury, amputations)	4.56	2.29	2.99	1.43	.000	1.03	5.45	2.51	3.07	1.48	.000	1.16
… a person with sexual problems/dysfunctions or concerns?	4.22	2.14	2.41	1.01	.000	1.08	5.44	1.99	2.85	1.54	.000	1.46
… a person with sexual problem(s) related to a medical, pharmacological, or surgical treatment	3.96	2.20	2.32	0.90	.000	0.98	5.42	2.06	2.92	1.58	.000	1.38
… a person whose gender and/or sex is different from your own	3.68	2.20	2.11	1.01	.000	0.92	4.07	2.16	2.12	1.19	.000	0.96
… a person whose gender is the same as your own?	2.47	1.09	1.53	0.80	.000	0.98	3.14	2.14	1.88	1.19	.000	0.73
… a person who is transgender or genderqueer	4.38	2.26	2.82	1.33	.000	0.84	5.03	2.68	2.97	1.42	.000	0.96
… a person who identifies as heterosexual	2.61	1.30	1.65	0.80	.000	0.89	3.19	2.14	1.94	1.28	.000	0.71
… a person who identifies as non-heterosexual (e.g., lesbian, gay, bisexual, something else)	3.42	1.70	2.18	1.00	.000	0.89	4.22	2.35	2.28	1.41	.000	1.00
… a person who identifies as asexual	4.12	2.64	2.89	1.86	.000	0.54	4.73	3.07	3.11	1.58	.000	0.66
… a person who engages in non-normative sexual practices (e.g., sadomasochism, paraphilias, or fetishes)	5.22	1.64	3.26	1.60	.000	1.21	5.42	2.13	3.37	1.65	.000	1.08
… a person who masturbates	3.74	2.22	1.77	0.98	.000	1.15	3.74	2.22	1.95	1.27	.000	0.99
… a person who engages in sex with a committed partner (i.e., dyadic relationship)	3.10	2.15	1.58	0.71	.000	1.01	3.19	2.15	1.79	1.09	.000	0.82
… a person who engages in casual sex (e.g., hook ups, one night stands)	2.73	1.33	1.73	0.69	.000	0.95	3.51	2.19	1.90	1.19	.000	0.91
… a person who engages in transactional sex (e.g., sex work, prostitution, etc.)	4.42	1.62	3.03	1.37	.003	0.93	4.96	2.24	2.75	1.34	.000	1.20
… a person who engages in sex with a person other than a partner in a dyadic relationship WITHOUT the other partner’s knowledge or consent (e.g., “cheating”)	4.65	1.88	2.93	1.42	.000	1.03	4.71	2.25	2.59	1.52	.000	1.10
… a person who engages in sex with a person other than a partner in a dyadic relationship WITH the other partner’s knowledge and consent (e.g., “open relationship”)	3.66	1.79	2.23	1.12	.000	0.96	4.38	2.29	2.17	1.29	.000	1.19
… a person who is coercive or abusive to their sexual partner(s)	5.51	2.57	4.34	2.12	.001	0.50	5.68	1.97	3.89	1.72	.000	0.97
… a person who is coerced or abused by their sexual partner(s)	4.54	2.61	3.23	1.34	.000	0.63	5.16	2.15	3.19	1.50	.000	1.62
… a person with questions about safer sex and sexually transmitted infections	5.52	1.90	1.67	0.85	.000	0.58	3.56	2.15	1.88	1.20	.000	0.96
… a person infected with the human immunodeficiency virus (HIV)	3.80	2.20	2.15	0.98	.000	0.97	4.88	2.19	2.34	1.28	.000	1.41
… a person who has a sexually transmitted infection OTHER than HIV	3.54	2.19	2.05	1.02	.000	0.87	4.75	2.18	2.19	1.24	.000	1.44
… a person who desires contraception	2.41	1.20	1.46	0.92	.000	0.89	3.26	2.17	1.77	1.25	.000	0.84
… a person who wishes to become pregnant or impregnate a partner?	2.71	1.29	1.74	0.94	.000	0.86	4.06	2.21	2.18	1.26	.000	1.05
… a person seeking an abortion?	3.71	2.41	2.12	1.98	.000	0.72	4.56	2.81	2.18	1.95	.000	0.98
… a person with conservative sociocultural beliefs about sexuality	3.71	1.39	2.30	1.14	.000	1.11	3.84	2.17	2.22	1.23	.000	0.92
… a person with liberal sociocultural beliefs about sexuality	2.90	1.36	1.84	0.83	.000	0.94	3.64	2.11	2.01	1.25	.000	0.94
… a person with religious/spiritual convictions about sexuality (in this context it refers to persons whose convictions stem from an organized religious group such as Catholicism, Islam, Judaism)	3.58	1.53	2.18	0.98	.000	1.09	3.79	2.58	2.38	1.20	.000	0.20
… a person who informs you of a topic that requires mandatory reporting (e.g., STI, threat of harm to others, etc.)	4.55	1.42	2.67	1.25	.000	1.41	4.82	2.62	2.62	1.23	.000	1.08
… a person whose values pertaining to one or several aspects of sexuality are in conflict with your own	3.93	1.54	2.45	0.96	.000	1.15	4.32	2.10	2.37	1.22	.000	1.14
… a person who requires referral for more specialized sexual healthcare	3.45	1.59	1.92	0.10	.000	1.19	4.59	2.53	2.23	1.29	.000	1.18

^a^Each question had responses which employed a 7-point Likert type scale in which 1 = very confident and 7 = very not confident. Higher mean scores reflect lower levels of confidence

^b^Mean on pretest, SD on pretest, Mean on post-test, SD on post-test

^c^Cohen’s *d* effect size: 0.20 small; 0.50 medium; 0.80 large; 1.40 huge

**Table 3:** Pre- and Post-Intervention Scores on the Attitude Subscale of the SHEPS

	Scores Pre- and Post-course^a^					
SHEPS Attitude Item	M*pre*^b^	SD*pre*	M*post*	SD*post*	*p*	*d*^c^
Educating teenagers on sex makes them more likely to do it.	6.01	1.20	5.93	2.15	.728	
Masturbation is a healthy part of human development	2.26	1.54	1.81	1.45	.000	0.30
Oral sex is an abnormal sexual practice	6.19	1.23	6.37	1.45	.273	
Anal sex is an acceptable sexual practice	2.80	1.84	2.09	1.58	.000	0.41
It is okay to have sex before marriage	1.80	2.08	1.46	2.42	.041	0.15
Marriage should be only between a man and a woman	5.70	3.03	5.64	3.01	.771	
I want to be a resource for my future patients with sexual problems	1.47	0.93	1.55	1.12	.602	
It is okay to have a non-monogamous relationship if both partners agree to it	2.09	1.99	2.32	1.84	.272	
It is not normal to be attracted to a person of the same sex	5.96	1.81	5.50	3.32	.260	
I won’t be able to provide care for patients with sexual problems	6.18	2.03	6.20	1.92	.931	
People who get sexual pleasure from inflicting and/or experiencing (sadomasochism) pain with consenting partners are sick	4.38	3.45	5.03	3.09	.071	
Abortion should be available to women for whatever reason they choose	2.11	2.10	1.99	2.17	.332	
Sex is not an issue that physicians should deal with in their practices	6.67	0.65	6.44	1.95	.331	
Abortion is only allowable in special cases (e.g. rape, incest, threat to health of mother)	5.70	2.22	5.40	2.98	.287	
Contraception should be easily available to anyone who wants it	1.29	0.87	1.23	0.64	.620	
Sexual problems (e.g. erectile dysfunction, low sex drive, pain with sex) are serious issues that should be addressed	1.79	1.05	1.38	0.89	.003	0.42
Being gay, lesbian, or bisexual is an acceptable lifestyle	1.75	1.58	1.44	2.29	.251	
Healthy women always have a lower sexual drive than men	5.53	3.12	6.33	1.25	.020	0.34
People who contract sexually transmitted infections get what they deserve	6.36	1.96	6.71	0.81	.137	
Abortion is murder	5.38	2.97	5.33	3.08	.867	
People who are transgender deserve to receive care to help them conform to their chosen gender	1.64	1.72	1.25	2.40	.080	
People should be allowed to marry someone of the same sex	1.41	1.83	1.42	1.69	.956	
All pornography should be banned	4.86	3.17	5.30	2.86	.340	
One can never be too old for sex	1.81	1.20	1.48	1.30	.016	0.31
I believe that being trained in human sexuality is important for health professionals.	1.30	0.57	1.26	0.55	.581	
I believe that I can use my human sexuality training effectively in a clinical setting.	1.49	2.35	1.81	1.19	.314	
I do not intend to use my human sexuality training in a clinical setting.	4.48	4.62	6.23	1.29	.002	0.52

^a^Each question had responses which employed a 7-point Likert type scale in which 1 = Strongly agree and 7 = Strongly disagree. Negative items were reversed so that the items are consistent in direction

^b^Mean on pretest, SD on pretest, Mean on post-test, SD on post-test

^c^Cohen’s *d* effect size: 0.20 small; 0.50 medium; 0.80 large; 1.40 huge: *d* only calculated for statistically significant *t* values

## Discussion

These longitudinal data evaluating a medical school sexual health course, using the SHEPS, are among the few recent systematic evaluations of such programs. Using the three domains of the SHEPS, covering self-reported communications skills, knowledge, and attitudes toward sexuality, it is evident (Table 2) that almost every item in the communications skills and knowledge domains significantly improved, and that the effect sizes in the pre-test to post-test are mostly in the large to huge effect size range using Cohen’s *d*. Clearly, the 20-h course, training, tutorials and OSCE positively impacted the communications skills and knowledge of the students in these two domains. OSCEs have been shown to have moderate inter-rater reliabilities, higher with experienced SPs [27]. However, the changes in attitudes about sexuality (Table 3) were few and small in effect, leading to the conclusion that attitudes about sexuality are minimally changed by a comprehensive and mandatory sexual course in first year medical students.

Several points stand out. Firstly, the 37 items in the first two scales were based on specific clinical scenarios of particular patient characteristics, so it is possible to determine which ones the course impacted most. For communication skills and knowledge, all those scenarios which were specifically addressed in the course, and those marginally or not addressed, were significantly improved. Those which were specifically addressed had the largest effect sizes. It seems, however, that the comfort with communications skills and knowledge generalized positively even in areas that were marginally addressed. As Zamboni and Ross [24] previously reported, “a rising tide lifts all boats”: comfort with communication skills in sexuality spread across sexual topics generally. Similarly, the increased knowledge in the knowledge domain of sexuality flowed into a wider spectrum of patient scenarios. That is, the knowledge about human sexuality was connectable and generalizable across these patient scenarios.

However, attitudes about sexuality changed minimally or if they did, the effect was small. This seems to fly in the face of the view from studies in the 1970s that SAR seminars were crucial for effective learning and patient care in the field of human sexuality. We believe that a more subtle interpretation of the evidence is called for. Firstly, we are 50 years past the so-called “sexual revolution” of the late 1960s and early 1970s. Sexuality is a common topic in the media and in public and private conversations, and the Internet makes sexual knowledge easily accessible. There is no longer much shock value in seeing explicitly sexual images and erotic videos online. We conjecture that it is likely that sexual attitudes are formed early, in adolescence and early adulthood, and that these students (median age 24) have formed and solidified their personal views about sexual issues in the decade before they come to medical school. Thus, there is minimal change or re-structuring of attitudes and beliefs at this point of early adulthood.

Second, the positive implication of these data is that medical students can learn effective communications skills and knowledge about human sexuality without attitudes interfering with that learning. The objective of the course was to give all medical students the skills and comfort to take a good sexual history without their attitudes and beliefs interfering with their skills, comfort or knowledge, not to change their attitudes. Such an interpretation is also supported by the lack of significant correlation between OSCE total scores and the Attitude scale total score, a measure of sexual health liberalism-conservatism. Third, we caution that the course did not include a SAR workshop as it is classically taught. It is difficult to replicate the intensity and interaction of a SAR and a small group experience in a large lecture theater, and it could be argued that nothing equivalent to a SAR (which includes sexually explicit media, exploring issues and concerns, and clarifying emotional response to aspects of sexuality) occurred [28]. SARs, however, are typically attended by a select sample, who are paying volunteers, and it may be regarded as inappropriate to require them of students in mandatory and for credit courses. Nevertheless, it is also possible that the SAR was a necessity of its time that now has less relevance, at least in the U.S., as a prerequisite for teaching about human sexuality.

Self-assessed communications and knowledge skills are difficult to verify independently. As part of completion of the course, there was an OSCE of 3 stations, each of 8 min of taking a sexual history, plus five minutes of feedback and discussion with the SP. We were able to incorporate the SP’s ratings into the data set. The literature suggests that there are few if any predictors of OSCE scores, since they test a viewpoint that is different from typical assessment measures. OSCEs are usually not significantly correlated with USMLEs or other assessments like multiple-choice questions [29, 30]. Violato [31] notes that they concentrate on skills, clinical reasoning, attitudes, and basic knowledge, but that they have had very little empirical scrutiny or validity.

As published findings indicated, communication skills and knowledge items were not associated with the OSCE score [27, 28]. This is consistent with the literature cited above which reports that OSCEs generally have few if any correlations with standard measures of performance, but evaluate a different set of skills. However, we did find (Table 3) that OSCE scores were significantly associated with 7 of the 26 items in the Attitudes subscale. Two of these predicted nearly a quarter of the variance in the OSCE score: believing that sex is not an issue that physicians should deal with in their practices, and believing that women always have lower sex drives than men. These data indicate that it may be attitudes about sexual health and its place in medical practice that influence OSCE performance, and not the skills and knowledge themselves. Positively, this may be interpreted as acknowledging that students can learn sexual health communications skills and knowledge and perform well in an OSCE without having their attitudes influence their performance.

It does not seem that the course as taught has much impact on attitude change. It may, however, also be that there are floor/ceiling effects on some items, especially if attitudes are already strongly held: item means clustered at high or low points of the scale support this. This would also be consistent with previously solidified attitudes with little opportunity to move. Further research on the relationships between healthcare student sexual health communications skills and attitudes about sexuality is warranted, as is debate on whether, at least in Western countries with wide discussion about sexuality and Internet access to erotica, the SAR has outlived its purpose.

This pre-test, post-test evaluation has strengths and weaknesses. A weakness is that the sample was selected based on willingness to fill in a questionnaire before and after the course, with a reasonable incentive, and while it did not differ from the composition of the course population based on sex, it was however not a full class sample. Adequate incentive rather than liking the content area should have reduced bias, especially as the questionnaire pre-test was several months before the course to minimize a course-enthusiasm recruitment effect. The final sample comprised a large proportion, 42.5% of the course. Using an instrument, the SHEPS, specifically designed and evaluated to assess sexual health educational interventions in healthcare professionals, and tested in both the U.S. and in Africa, was a strength. However, we caution over-generalization of these data beyond a large Midwest state U.S. medical school.

## Conclusions

We believe that this study adds to the sparse and somewhat dated body of literature on longitudinal evaluation of the impact of comprehensive sexual health courses in healthcare students. The utility of the SHEPS as a tool for evaluation of sexual health education in healthcare professionals is excellent, for identifying specific content areas of strength or weakness rather than just general impressions, and to calculate effect sizes as well as significance. However, confidence in dealing with patients with a variety of presenting sexually-related issues, many of which were not explicitly taught in the course, suggests generalization to more other sexuality-related issues. While we evaluated an entire course, it is possible to use the SHEPS at several points during a course and to identify where and how changes in curricula are occurring. It this study, we have also demonstrated that changing sexual attitudes is not necessarily a prerequisite to teaching medical students the skills and knowledge to comfortably (for both provider and patient) take a thorough and comprehensive sexual history.

## Data Availability

The datasets generated and analyzed during the current study are not publicly available, due to being course evaluations containing student educational data including performance, under FERPA (the US Family Educational Rights and Privacy Act).

## References

[1] Shindel AW, Parish SJ (2013). Sexuality education in North American medical schools: current status and future directions. J Sex Med.

[2] Coleman E, Elders J, Satcher D, Shindel A, Parish S, Kenagy G (2013). Summit on Medical School Education in Sexual Health: Report of an Expert Consultation. J Sex Med.

[3] Criniti S, Andelloux M, Woodland MB, Montgomery OC, Hartmann SU (2014). The State of Sexual Health Education in U.S. Medicine. Am J Sex Ed.

[4] Shindel AW, Baazeem A, Eardley I, Coleman E (2016). Sexual health in undergraduate medical education: Existing and future needs and platforms. J Sex Med.

[5] Eardley I, Reisman Y, Goldstein S, Kramer A, Dean J, Coleman E (2017). Existing and future educational needs in graduate and postgraduate education. J Sex Med.

[6] Solursh D, Ernst J, Lewis RW (2003). The human sexuality education of physicians in North American medical schools. Int J Impot Res.

[7] Capiello J, Coplon L, Carpenter H (2017). Systematic review of sexual and reproductive health content in nursing curricula. J Obstet Gynecol Neonatal Nurs.

[8] Malhotra S, Khurshid A, Aff MP, Hendricks KA, Mann JR (2008). Medical School Sexual Health Curriculum and Training in the United States. J Natl Med Assoc.

[9] Marzano RJ (2001). Designing a New Taxonomy of Educational Objectives. Experts in Assessment.

[10] Parish SJ, Clayton AH (2007). Sexual medicine education: Review and commentary. J Sex Med.

[11] Marcotte DB, Logan C (1977). Medical sex education: Allowing attitude alteration. Arch Sex Behav.

[12] Golden J, Liston E (1972). Medical sex education: The world of illusion and the practical realities. J Med Ed.

[13] Marcotte DB, Kilpatrick D (1974). Preliminary evaluation of a sex education course. J Med Ed.

[14] Garrard J, Vaitkus A, Held J (1976). Follow-up Effects of a Medical School Course in Human Sexuality. Arch Sex Behav.

[15] Miller WR, Lief HI (1979). The sex knowledge and attitude test (SKAT). J Sex Mar Ther.

[16] Lief H, Davis CM, Yarber WL, Bauseman R, Schreer G, Davis SL (1998). The Sexual Knowledge and Attitudes Test. Handbook of Sexuality Measures.

[17] Schnarch DM, Jones K (1981). Efficacy of sex education courses in medical school. J Sex Mar Ther.

[18] Leiblum SR (2001). An established medical school human sexuality curriculum: Description and evaluation. Sex Relat Ther.

[19] Rosen R, Kountz D, Post-Zwicker T (2006). Sexual communication skills in residency training: The Robert Wood Johnson model. J Sex Med.

[20] Ross MW, Channon-Little LD (1991). Discussing Sexuality: A Guide for Health Practitioners.

[21] Ross MW, Channon-Little LD, Rosser BRS (2000). Sexual Health Concerns: Interviewing and History Taking for Health Practitioners.

[22] Roth Bayer C, McKool M, Shindel A (2018). Sexual Health Education for Professionals Scale (SHEPS) in Ross et al. Evaluation of an assessment instrument for a sexual health curriculum for nurses and midwifery students in Tanzania: The Sexual Health Education for Professionals Scale (SHEPS). Appl Nurs Res.

[23] Ross MW, Leshabari S, Rosser BRS, Trent M, Mgopa L, Wadley J, Kohli N, Agardh A (2018). Evaluation of an assessment instrument for a sexual health curriculum for nurses and midwifery students in Tanzania: The Sexual Health Education for Professionals Scale (SHEPS). Appl Nurs Res.

[24] Zamboni BD, Ross MW (2020). Measuring the effect of sexual health education training in professionals: Structure of the Sexual Health Education for Professionals Scale (SHEPS) among marriage and family therapy graduate students. J Mar Fam Ther.

[25] Ross MW, Ayers J, Schmidt W (2019). A new computer application for teaching sexual history taking to medical students: innovation and evaluation in the UfaceME® program. Adv Med Ed Prac.

[26] Effect size calculator for t-test. https://www.socscistatistics.com/effectsize/default3.aspx Viewed 8/16/2020.

[27] Mortsiefera A, Kargerb A, Rotthoff T, Raskid B, Pentzeka M (2017). Examiner characteristics and interrater reliability in a communication OSCE. Pat Ed Couns.

[28] Rosser BRS, Dwyer M, Coleman E, Miner M, Metz M, Robinson BE, Bockting WO (1995). Using sexually explicit material in adult sex education. J Sex Ed Ther.

[29] Dong T, Saguil A, Artino AR (2012). Relationship between OSCE scores and other typical medical school performance indicators: A 5-year cohort study. Mil Med.

[30] Park WB, Kang SH, Lee YS (2015). Does objective structured clinical examination score reflect the clinical reasoning ability of medical students?. Am J Med Sci.

[31] Violato C (2019). Assessing competence in medicine and other health professions.

